# Numerical Simulation of Galvanic Corrosion and Electrical Insulation for TC4/304 Galvanic Couple

**DOI:** 10.3390/ma18010038

**Published:** 2024-12-25

**Authors:** Kaixun Liu, Yuhang Wu, Huicong Liu, Haining Chen, Weiping Li

**Affiliations:** School of Materials Science and Engineering, Beihang University, Beijing 100191, China

**Keywords:** galvanic corrosion, electrical insulation, finite element, simulation, titanium

## Abstract

The galvanic corrosion and electrical insulation between TC4 Ti-alloy and 304 stainless steel coupled in pipe joints were investigated using the finite element method. The results obtained from polarization were applied as boundary conditions. The simulation incorporated secondary current distribution with chemical species transport and laminar flow. COMSOL modeling provided calculated values that showed good agreement with experimental measurements. The model was utilized to examine the influence of geometric and environmental factors, including insulation resistance, insulation distance, pipe diameter, temperature, and electrolyte concentration, on the galvanic corrosion process. The results indicated that electrical insulation performance significantly affects the corrosion process.

## 1. Introduction

In maritime engineering, the utilization of a diverse range of pipework systems is important aboard ships. The choice of pipe materials hinges upon several pivotal factors, such as location, operating conditions, and budgetary considerations. It is commonplace in ship pipeline design to incorporate dissimilar metal pipelines. Flanges serve as pivotal components, facilitating the connection of pipes by bolting two flanges together with spacers in between. To mitigate electrochemical corrosion between dissimilar metal pipelines or flanges, insulating spacers are typically incorporated into the initial design to isolate distinct metal components [1]. The adoption of electrical insulation measures can effectively mitigate galvanic corrosion between the steel ship hull and titanium alloy [2]. Polymer insulation applied to the bolted joint between AZ31B and carbon fiber-reinforced composite has been shown to significantly reduce galvanic corrosion [3]. However, galvanic attack may still occur due to the permeation of an aqueous corrosive medium. As service time increases, the insulating properties of these spacers deteriorate, accelerating the corrosion of one of the coupled dissimilar metals.

Numerous experimental and simulation-based studies [4,5,6,7,8,9,10,11,12,13] have investigated the effects of various factors on galvanic corrosion, such as potential difference, anode/cathode area ratio [14], distance between cathode and anode, temperature [15], and flow rate [16]. The main conclusions from these studies are summarized as follows. The galvanic current density of the anode increased with the decrease of the anode/cathode area ratio. The galvanic current density and average galvanic current of the anode decreased as the distance between the cathode and anode increased. The corrosion rate became higher with the temperature increased. Zhang et al. [17] studied the galvanic corrosion behavior in a simulated pipeline system. Their study found that the galvanic couple comprising titanium and high-strength steel exhibited an increased current density, which was further amplified with rising temperature and a larger cathode-to-anode area ratio.

However, limited research has been focused on the role of insulating spacers between the anode and cathode in these systems. The increase in the insulating distance results in a longer circuit path, thereby elevating the resistance of the circuit. Song [18] investigated the coupling of magnesium alloy AZ91D with aluminum alloy, stainless steel, and zinc. Their findings showed that the current is non-linearly dependent on spacer width under salt spray conditions, with the current decreasing as the thickness of the insulating gasket increases. Notably, the rapid decrease in current is observed within the first 1 cm, while the decreasing trend slows as the thickness increases further. When the spacer thickness reaches 9 cm, the corrosion current is 22μA/cm^2^. The results suggest that while insulating spacers can increase the resistance of the aqueous film on the surface, they may not completely block the electrical path. Simulations have been used effectively to analyze potential and current distributions, providing insights into sensitive areas for galvanic corrosion and estimating its rate [19]. However, most models tend to overlook the effects of resistance and the limitations of mass transfer [20]. Therefore, additional studies are required to incorporate these factors into simulation models to better understand and mitigate galvanic corrosion.

Pan et al. [21] have tested the corrosion potential and current in seawater by connecting resistors with different resistance values between commercially pure titanium TA2 and high-strength steel as well as copper-nickel alloy B10 and high-strength steel and found that the galvanic couples were nearly insulated and the extent of galvanic corrosion was controlled significantly when the galvanic insulation value between the two materials was higher than 10 kΩ and 50 kΩ. Similarly, Zhang et al. [22] have investigated galvanic current density in both static and dynamic seawater environments by connecting different insulated resistances between TA2 and B10 and found that the galvanic corrosion could be avoided when the connecting resistance was higher than 56 kΩ. The performance of electrical insulation between dissimilar metals is directly associated with the resistance of the insulating spacer, but few related models have been reported.

It is crucial to promptly identify the relationship between insulation properties and corrosion behavior and to monitor the electrical insulation between dissimilar metals, especially in real-world environments where corrosion sites extend beyond pipe joints. The corrosion phenomenon is challenging to reproduce, so this model was developed to address the issue and to study the impact of relevant factors. This paper investigates the effect of electrical insulation properties on corrosion behavior, including self-corrosion and galvanic corrosion, by modeling electrical insulation control and corrosion behavior, which can provide a foundation for effective electrical insulation control.

## 2. Materials and Methods

### 2.1. Materials

**Figure 1:**
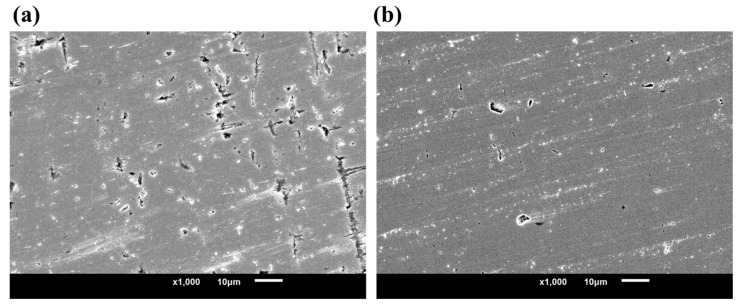
SEM (**a**) TC4 surface; (**b**) 304 surface.

TC4 Ti-ally and 304 stainless steel were used in this study, which is widely used in pipework systems [23]. The TC4 titanium alloy samples were sourced from Xinghua Zengyitai Co., Ltd., Xinghua, China, while the 304 stainless steel samples were obtained from Tianjin Xingang Co., Ltd., Tianjin, China. The chemical compositions from the manufacturer’s report are shown in Table 1 and Table 2. The specimens were processed into 110 mm × 25 mm × 5 mm and 55 mm × 25 mm × 5 mm plates. The surfaces are shown in Figure 1a,b.

### 2.2. Polarization and Conductivity Measurement Experiment

Polarization curves of the specimens were measured using a four-channel electrochemical workstation from the Wuhan Corr Test. Before the experiment, the specimens were polished with water-abrasive sandpaper in the order of #240, #600, #800, and #1000 so that no processing traces were basically visible on the surface of the specimens. A three-electrode system was used to measure the polarization curves of stainless steel and titanium alloy specimens, which specimen acted as the working electrode. The platinum and standard calomel electrodes were used as counter electrodes and reference electrodes, respectively. Initially, the open-circuit potentials were measured using an electrochemical workstation, and the stabilized open-circuit potential was recorded. Subsequently, the polarization curves were measured. The conductivity of the electrolyte solution was tested by using a Lei-ci DDS-307A conductivity tester from Shanghai Yidian Co., Ltd., Shanghai, China. An electrolyte solution of 3.5 wt.% NaCl was used in this study. The measurements were conducted at 25 °C.

### 2.3. Corrosion Test

The stainless steel specimen and titanium alloy specimen were coupled. Two samples are insulated from each other, with the spacing adjustable and ranging from 5 mm to 1 cm, 5 cm, or 10 cm. The surface area of both sides of the test site was 12.5 cm^2^. When measuring with the electrochemical workstation, the TC4 titanium alloy specimen was connected as the working electrode, the mercuric electrode was connected to the reference electrode, and the 304 stainless steel was grounded. In the corrosion test, the galvanic corrosion test was chosen. The test interval was set to 50 s, with a total test duration of 10,000 s. Throughout the test, the corrosion current and corrosion potential curves of the galvanic couple were recorded over time. The corrosion test was conducted according to the standard “HB 5374-1987 [24] Method for Measuring Galvanic Current of Different Metals”.

### 2.4. Numerical Model and Boundary Conditions

**Figure 2:**
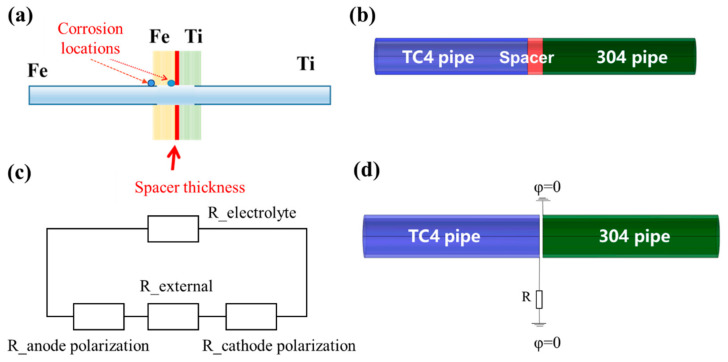
Details of model. (**a**) Diagram of galvanic corrosion; (**b**) diagram of TC4/304 pipe couple; (**c**) equivalent circuit model; (**d**) diagram of the model.

COMSOL modeling was established for the galvanic corrosion test and electrical insulation control experiment to simulate the corrosion of the two test couples. The model was solved using COMSOL V6.1. In the electrical insulation control experiment, the insulation spacers were placed between two pipes. One terminal with an external short-circuit resistance R was set to connect to the ground, and the potential of the other terminal was set to zero. As depicted in Figure 2b, the blue part on the left represents the titanium alloy pipe, and the green part on the right represents the stainless steel pipe. The equivalent circuit diagram of the galvanic corrosion model is shown in Figure 2c [15]. External resistance includes resistance of the solution between the anode and cathode. As depicted in Figure 2d, the schematic diagram of the simulation concept illustrates that the titanium alloy boundary was grounded after connecting an external short-circuit resistance, while the potential at the stainless steel pipe boundary was set to zero. At this point, the coupling resistance between the two pipes can be adjusted.

The secondary current distribution with chemical species transport and laminar flow were considered for simulation. The insulation boundary does not allow current to flow through. The conductivity is constant, the electrolyte composition remains near-constant, and the current density is limited by the mass transport of the electroactive species. The current density can be calculated using the sum of the fluxes of all ions. The transport of ionic species can be described by the Nernst–Planck equation [6,25,26]. The charge transfer in the electrolyte is also assumed to obey Ohm’s law [27].

(1) $$\overset{⃑}{l_{l}}=F\sum_{i=1}^{n}z_{i}\left(-D_{i}\nabla c_{i}-z_{i}u_{i}Fc_{i}\nabla \phi_{l}+c_{i}\vec{u}\right)=-\sigma_{l}\nabla \phi_{l}$$

where $\overset{⃑}{l_{l}}$ (A/m^2^) denotes the current density vector in the electrolyte, $\sigma_{l}$ (S/m) denotes the conductivity, ${\nabla \phi }_{l}$ (V/m) denotes the electrolyte potential gradient, *F* (96,485 C/mol) denotes the Faraday constant, $z_{i}$ (1) represents charge number, $D_{i}$ (m^2^/s) is the diffusion coefficient, $u_{i}$ (s·mol/kg) is the mobility, $c_{i}$ (mol/m^3^) is the concentration of the ion and $\vec{u}$ (m/s) is the velocity vector.

The current density due to the electrochemical reactions is also described as a function of the overpotential in the electrode surface. The current density is given by Equation (2). The overpotential *η* (V) is given by Equation (3) [27,28].

(2) $$-\vec{l_{s}}\cdot \vec{n}=\vec{l_{l}}\cdot \vec{n}=\sum {(i}_{0}(c_{R}\exp\left(\frac{\alpha_{a}F\eta }{RT}\right)-c_{O}exp(\frac{-\alpha_{c}F\eta }{RT})))$$

(3) $$\eta =\phi_{s}-\phi_{l}-E_{eq}$$

where $\overset{⃑}{l_{s}}$ (A/m^2^) denotes the current density vector in the electrode, $\vec{n}$ is the normal vector, $c_{R}$ (1) and $c_{O}$ (1) are related to the concentration of reducers and oxidizers, and $\alpha_{a}$ (1) is anodic transfer coefficient. $\alpha_{c}$ (1) is cathodic transfer coefficient. *R* (8.314 (J/(mol·K)) denotes the gas constant, *T* (K) denotes the temperature, $\phi_{s}$ (V) is the electrode potential, and $E_{eq}$ (V) is the equilibrium potential of the reaction.

**Figure 3:**
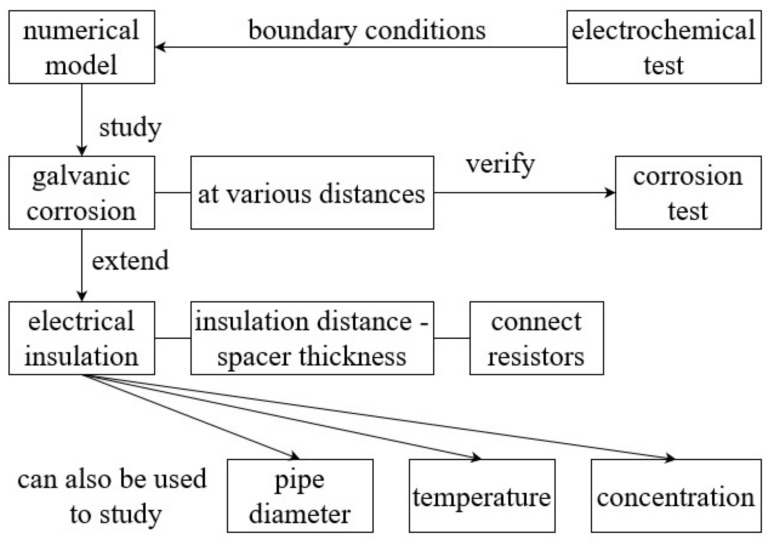
Flowchart.

The flowchart of the simulation process is given in Figure 3. When investigating the influence of various factors, the relevant parameters are systematically varied while all other parameters remain constant. Electrochemical parameters are detailed in the results. The parameter setting is listed in Table 3.

## 3. Results

### 3.1. Electrochemical Test Results

**Figure 4:**
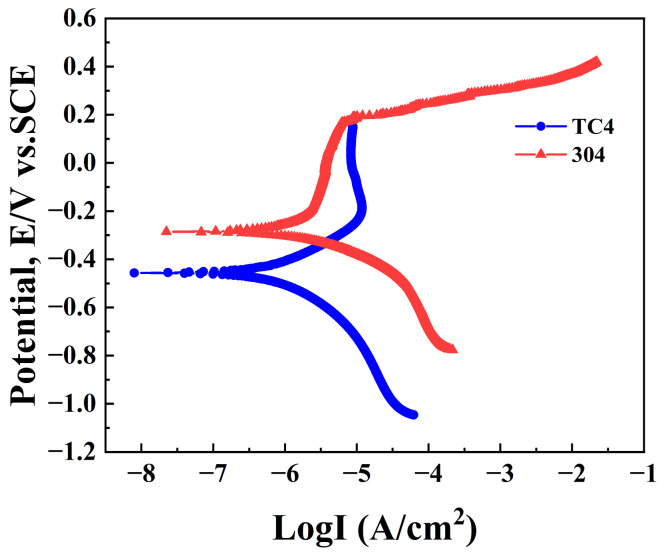
Tafel curve for TC4 and 304 in 3.5 wt.% NaCl solution at 25 °C.

The measured conductivity of 3.5 wt.% NaCl solution is 5.6 S/m at 25 °C. The Tafel polarization curves were plotted against the potential versus logarithmic current density. The polarization curves obtained for TC4 and 304 are shown in Figure 4. The values of equilibrium potential, exchange current density, anodic Tafel slope, and cathodic Tafel slope were obtained from the graph. The results obtained for TC4 and 304 are shown in Table 4. The results obtained from polarization were applied as boundary conditions. The corrosion potential of TC4 is lower than that of 304.

**Figure 5:**
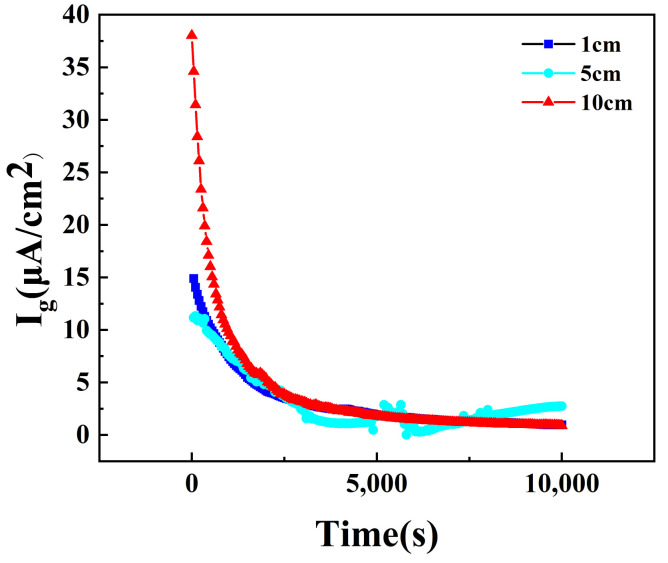
Corrosion current over time in 3.5 wt.% NaCl solution at various anode and cathode distances.

The curves of the corrosion current of different anode and cathode distances obtained through experimental testing over time are shown in Figure 5. To determine the experimental test value of the galvanic current for comparison with the simulated results of galvanic corrosion as the distance between the anode and cathode varies, the average stabilized galvanic current is calculated.

Based on the analysis of mixed potential prediction theory, it is understood that when TC4 forms a galvanic couple with 304, the self-corrosion potential of TC4 is more negative. The dissolution and corrosion rates of TC4 are accelerated under anodic polarization. Conversely, the relatively positive free corrosion potential of 304 causes it to act as the cathode, thereby suppressing the corrosion of 304.

### 3.2. Simulation Results

#### 3.2.1. Simulation Results of Galvanic Corrosion at Various Distances

**Figure 6:**
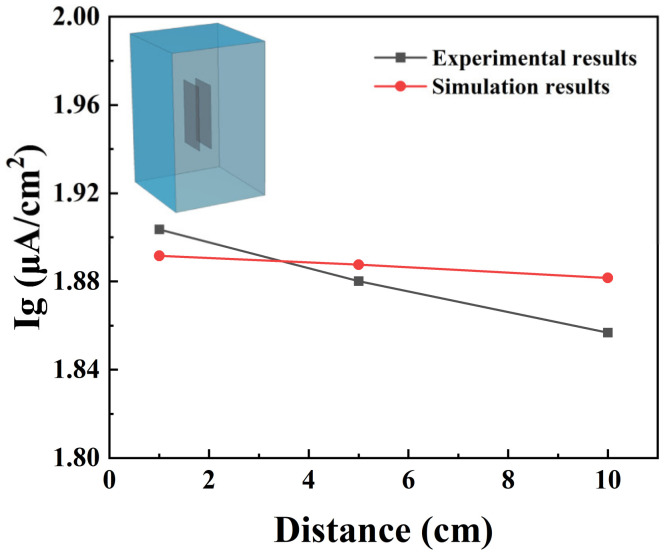
Simulation results and experimental results of corrosion current at various distances.

COMSOL modeling provided calculated values that showed good agreement with experimental measurements. The simulation results and experimental results of corrosion current at various distances are shown in Figure 6. The errors between the simulated results and the experimental results are 0.65%, 0.4%, and 1.32%. The electrochemical field, flow field, and material transfer field are coupled, and the galvanic corrosion model results incorporate concentration polarization and exhibit greater alignment with experimental test findings. Moreover, the model takes into account a more comprehensive range of influencing factors. When the distance is set to 5 cm, the error is minimized. Within the permissible error range, the predicted current density can be used to describe the experimental results.

#### 3.2.2. Effect of Electrical Insulation of Anode/Cathode

**Figure 7:**
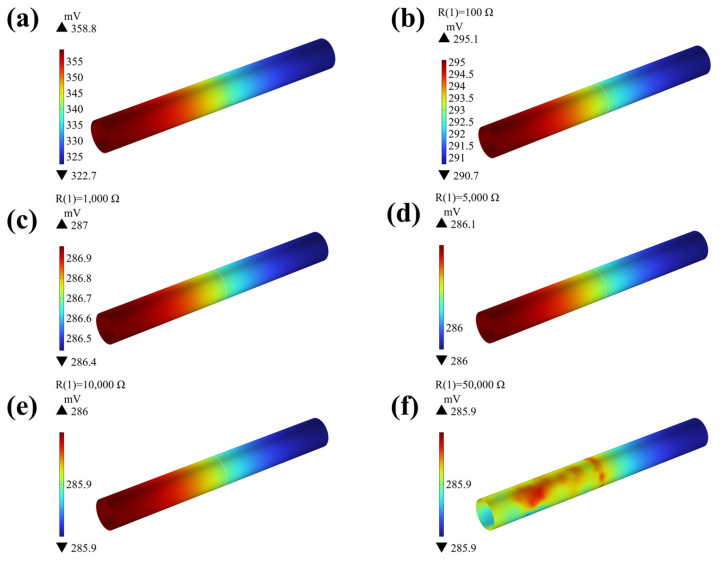
The opposite surface potential distribution of pipe in direct contact and connection with different resistance values: (**a**) 0; (**b**) 100 Ω; (**c**) 1000 Ω; (**d**) 5000 Ω; (**e**) 10,000 Ω; (**f**) 50,000 Ω.

The numerical simulations of galvanic corrosion on pipeline models involve direct coupling and are connected at different resistance values. The surface potential distribution of pipe in direct contact and connection with different resistance values are shown in Figure 7. Analysis of the potential distribution reveals that direct contact results in a negative coupling potential, with the anode pipe end exhibiting the most negative potential and the cathode pipe end showing the most positive potential. The maximum difference in coupling potential can reach approximately 30 mV, demonstrating a relatively uniform distribution of coupling potential.

Upon connecting resistors, it is observed that increasing resistance values result in a gradual shift towards positive coupling potentials. For instance, when the simulated resistance value reaches 1000 Ω, the coupling potential approaches −286 mV. Subsequent increases in simulated resistance do not significantly alter the coupling potential, which remains below 1 mV. Furthermore, the difference in coupling potential between pipeline sections diminishes as resistance values rise. Notably, compared to the initial 30 mV coupling potential difference at a connection resistance of 100 Ω, the disparity becomes negligible when the simulated resistance exceeds 1000 Ω. This decreasing trend in galvanic corrosion propensity is evident with increasing resistance.

At a connection resistance of 50 kΩ, a significant shift in the potential distribution of the pipeline occurs. In the anode section on the left, the potential distribution is no longer ordered from low to high, and the most severely corroded segment is no longer the electrical connection point. This transition is attributed to the substantial resistance value of 50 kΩ, which inhibits galvanic corrosion on the titanium alloy pipeline, leading primarily to self-corrosion. Consequently, potential polarization across various pipeline positions becomes more uniform, deviating from the previous axial distribution pattern along the pipeline’s length.

**Figure 8:**
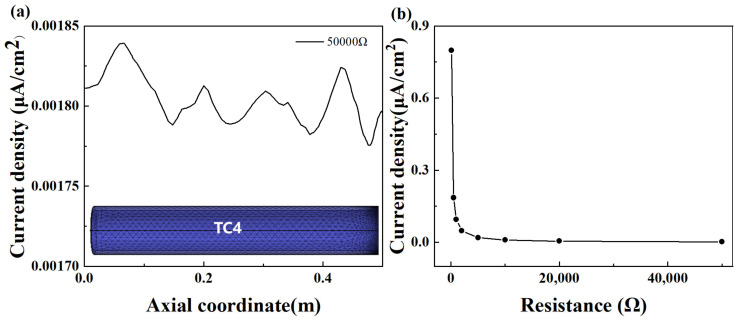
(**a**) The surface current density distribution of TC4 pipe in the electrolyte at a connection resistance of 50,000 Ω; (**b**) maximum current density with different connection resistance values.

The current density increases along the pipe from both ends toward the center, peaking at the connection point. Within a specific range, an increase in connection resistance results in a corresponding decrease in current density. Upon reaching a connection resistance of 50 kΩ, the current density distribution on the TC4 pipeline becomes uneven, and the maximum current density is no longer observed at the connection point, as shown in Figure 8a. The maximum galvanic corrosion current density varies with the connection resistance, as illustrated in Figure 8b. It is evident that as the resistance value increases, the corrosion current density diminishes. When the resistance surpasses 500 Ω, the corrosion current density falls below 0.3 μA/cm^2^, thereby achieving effective insulation control for galvanic corrosion.

#### 3.2.3. Effect of Insulation Distance

**Figure 9:**
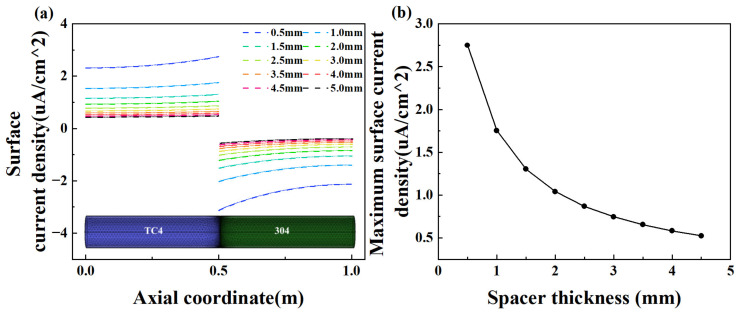
(**a**) The current density distribution along the TC4 and 304 surfaces in the electrolyte with various spacer thicknesses; (**b**) maximum current density with different spacer thicknesses.

The effect of thickness of the insulating spacer thickness between TC4 and 304 on galvanic corrosion is shown in Figure 9. The typical spacer thickness ranges from 1.5 mm to 3 mm. The investigation has been extended to include a range from 0.5 mm to 5 mm. As the spacer thickness increases, the connection resistance correspondingly increases. As the thickness increases from 0.5 mm to 5 mm, the current density decreases, consistent with the current density trend observed in the preceding section. In our design, increasing the spacer thicknesses results in a corresponding increase in connection resistance. It was assumed that the resistance and distance exhibit a linear relationship. The increase in the insulating distance results in a longer circuit path, thereby elevating the resistance of the circuit. Consequently, the impact of increasing the spacer thickness is analogous to that of raising the resistance. The peak anodic current density is non-linearly dependent on spacer width [18].

#### 3.2.4. Effect of Pipe Diameter

**Figure 10:**
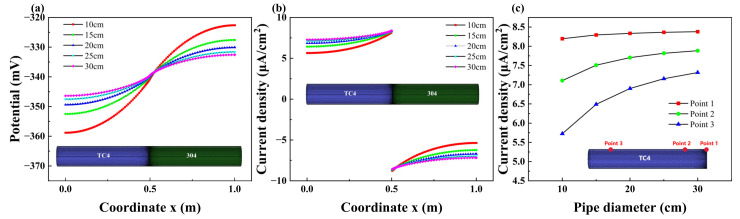
Variations of potential (**a**) and current density (**b**) with pipeline diameter; (**c**) variations of current density with pipe diameter at different points.

Galvanic corrosion simulations were conducted on pipes with five different diameters, ranging from 10 cm to 30 cm in 5 cm intervals, while other conditions were kept constant. Figure 10 illustrates the axial variation curve of galvanic corrosion current density for these different pipe diameters. It is evident that the current density increases gradually with the enlargement of the pipe diameter, leading to an intensified degree of corrosion. Additionally, as the pipe diameter increases, the galvanic corrosion potential of the TC4 pipeline shifts positively, while the potential of the 304 pipeline shifts negatively due to the hindrance of the corrosion reaction during the cathodic process.

Pipe surface current density increases gradually with the expansion of pipe diameter from 10 cm to 30 cm. This can be attributed to the generation of an electric field in the electrolyte solution resulting from galvanic corrosion within the pipeline. The pipeline subsequently exhibits a shielding effect on the electric field [4]. As the pipe diameter increases, the shielding effect decreases, leading to an increase in the galvanic corrosion current density. Analysis of various calculation points along the TC4 pipeline reveals that the current density at point 3 demonstrates the most substantial variation in response to changes in pipe diameter. In contrast, the variation in current density at point 1, which is located near the connection, is relatively minor when subject to fluctuations in pipe diameter. This observation suggests that changes in pipe diameter significantly impact galvanic corrosion in areas that are distant from the pipe connection.

#### 3.2.5. Effect of Temperature

**Figure 11:**
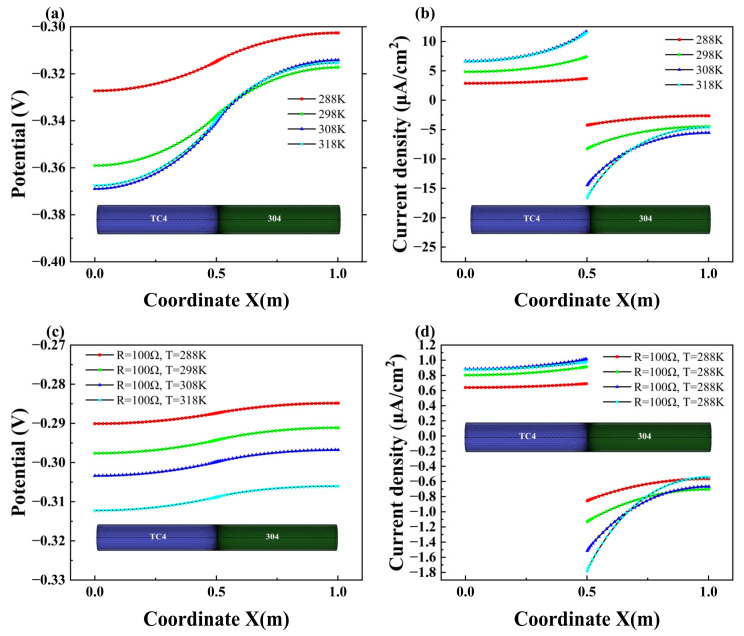
Variations of potential (**a**) and current density (**b**) in direct contact at different temperatures; variations of potential (**c**) and current density (**d**) at a connection resistance of 100 Ω at different temperatures.

The influence of temperature on the galvanic corrosion process is reflected in its impact on electrical conductivity and the electrochemical kinetics equation. Figure 11a,b illustrate the axial distribution of potential and current density during the corrosion process of the pipe in direct contact at different temperatures. It can be observed that as the temperature increases, the polarization value of the anode (TC4) decreases while the polarization value of the cathode (304) increases, leading to an increase in the corrosion current density. In the galvanic corrosion process, elevated temperature enhances cathodic polarization, resulting in an increase in current density.

When a resistance is connected between the pipelines, the corrosion behavior of the pipelines varies with temperature, as shown in Figure 11c,d. It is evident from the graph that when a 100 Ω resistance is connected, the influence of temperature on the potential variation across different parts of the anode becomes more uniform from Figure 11a,c. Compared to the unconnected resistance scenario, the distribution of potential becomes more uniform. Particularly near the connection, the effect of temperature on the galvanic corrosion current density significantly decreases after connecting the resistance.

As the temperature increases, the anode potential becomes more negative, which results in higher current density for the anode (TC4). The current density increases correspondingly as the temperature increases from 288 K to 308 K. However, the difference between the results at 308 K and 318 K is minimal, as the influence of temperature on the equilibrium potential becomes more pronounced. The impact of temperature on the equilibrium potential should be considered in future research.

#### 3.2.6. Effect of Electrolyte Concentration

**Figure 12:**
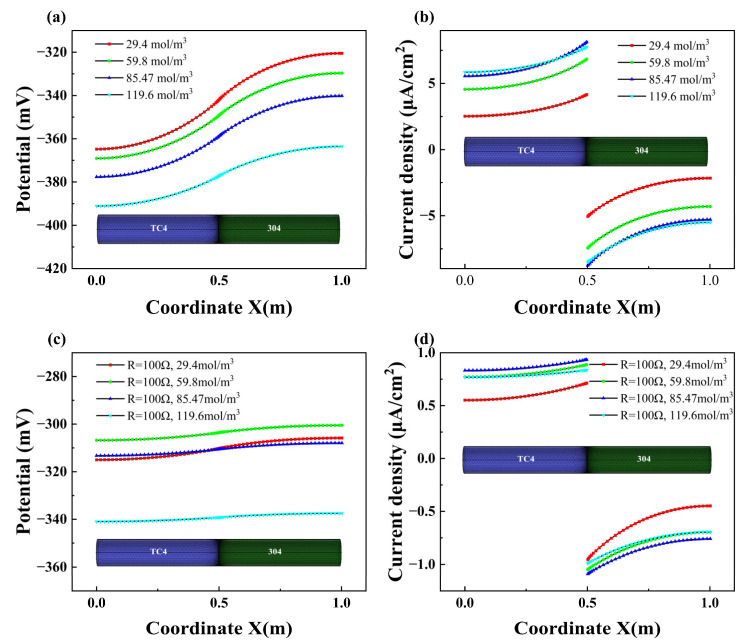
Variations of potential (**a**) and current density (**b**) in direct contact at different electrolyte concentrations; variations of potential (**c**) and current density (**d**) at a connection resistance of 100 Ω at different electrolyte concentrations.

The influence of concentration on the galvanic corrosion process is reflected in its effect on conductivity and the behavior of cathodic and anodic corrosion. Figure 12 presents the simulation results of TC4/304 couple corrosion models at varying concentrations. The figure illustrates that as the concentration increases, the anode potential becomes more negative, which leads to a higher current density of the anode (TC4). The current density increases as the concentration rises from 29.4 mol/m^3^ to 85.47 mol/m^3^. However, the minimal variation between the results at 85.47 mol/m^3^ and 119.6 mol/m^3^ indicates that the replenished electrolyte is sufficient to compensate for the consumption.

The potential polarization value of the anode pipeline (TC4) decreases as the concentration increases, whereas the polarization value of the cathode pipeline (304) continues to increase. This disparity is attributed to the greater influence of concentration on cathodic polarization behavior compared to anodic polarization. Analysis of the potential and current density distribution curves of pipelines in the TC4/304 couple corrosion model with different concentrations reveals a similar effect of concentration across various sections of the TC4/304 pipeline. With increasing concentration, the magnitude of potential and current changes, both near and far from the connection, remains relatively uniform. This uniformity can be attributed to the fact that the increase in concentration in the galvanic corrosion model of the pipeline primarily governs the polarization of galvanic corrosion as influenced by concentration. Consequently, when the concentration distribution of the pipeline at different concentrations displays minimal variance, the corrosion disparities among different sections are also insignificant. When a 100 Ω resistance is connected, Figure 12c,d depict the current density and potential distribution on the surface of the pipeline at different electrolyte concentrations. The addition of the connecting resistor leads to a reduction in the current density on the pipeline surface. Comparative analysis of the results obtained without adding a resistor shows that after connecting the resistor, the variation in galvanic corrosion current density, especially further from the connection, diminishes with the change in electrolyte concentration.

## 4. Conclusions

To effectively explain pipeline corrosion, a model was constructed to reproduce the conditions leading to corrosion. The model parameters were derived from polarization and conductivity testing. Once the initial model was established, electrochemical corrosion behavior was verified through experiments, enabling the model to be used for calculating corrosion parameters. The model was extended to consider the influence of various factors on galvanic corrosion in pipelines. The model not only enhances our understanding of the galvanic corrosion mechanism but also predicts the location and current density, providing valuable insights for future structural optimization and electrical insulation control against galvanic corrosion.

1. The numerical simulation model for TC4/304 galvanic corrosion was successfully developed and validated against experimental results. The discrepancy between the simulated galvanic corrosion current and the experimentally measured galvanic current ranges from 0.4% to 1.32% as the distance between the anode and cathode varies. This model was utilized to investigate the influence of geometric and environmental factors on galvanic corrosion.
2. Within a specified range, an increase in insulation resistance resulted in a reduction of the potential difference between the anode and cathode, thereby decreasing the occurrence of galvanic corrosion. Upon reaching a sufficiently high level, the maximum current density at the connection decreased, potentially leading to self-corrosion. The tendency for galvanic corrosion decreases significantly with increasing resistance. When the resistance exceeds 500 Ω, the corrosion current density falls below 0.3 μA/cm^2^, effectively achieving insulation control over galvanic corrosion. As the resistance surpasses 1000 Ω, the difference in coupling potential becomes negligible becomes negligible. Furthermore, when resistance reaches 50,000 Ω, the phenomenon of uneven potential distribution becomes distinctly observable.
3. The surface current density of the pipe increases gradually as the pipe diameter expands from 10 cm to 30 cm. Changes in pipe diameter significantly impact galvanic corrosion in areas distant from the pipe connection. The current density increases correspondingly with rising temperature, from 288 K to 308 K. Similarly, as the concentration rises from 29.4 mol/m^3^ to 85.47 mol/m^3^, the current density also increases. However, while the value continues to rise, the variation in current density becomes minimal. Further research will be conducted to investigate the effects of temperature on equilibrium potential, as well as the influence of flow on concentration and conductivity. The introduction of resistance resulted in a deceleration of the changes in potential and current density concerning temperature and concentration.

## Figures and Tables

**Table 1:** Chemical composition of the TC4 Ti-alloy (mass%).

Alloy	Al	V	Fe	Si	C	N	H	O
C4	6.2	4	0.3	0.15	0.1	0.05	0.015	0.2

**Table 2:** Chemical composition of the 304 stainless steel (mass%).

Alloy	C	Si	Mn	S	P	Cu	Co	B	Ni	Cr
304	0.035	0.66	1.88	0.005	0.023	1	0.06	0.0018	9.27	18.65

**Table 3:** COMSOL model parameters.

Parameters	Value	Unit
*d*	0.0015	m
**T**	298	K
*D*	1 × 10^−9^	m^2^/s
*c_in_*	59.8	mol/m^3^
*u_in_*	1 × 10^−4^	m/s
*c_0_*	59.8	mol/m^3^

**Table 4:** Results of Tafel polarization in 3.5 wt.% NaCl solution at 25 °C.

Sample	Equilibrium Potential	Exchange Current Density	Anodic Tafel Slope	Cathodic Tafel Slope
TC4	−0.4842 V	0.0375 A/m^2^	0.4667 V/dec	
304	−0.2218 V	0.0393 A/m^2^		−0.1718 V/dec

## Data Availability

The data presented in this study are available upon request from the corresponding author.

## Nomenclature

**Symbology**	**Description**	**Unit**
$\overset{⃑}{l_{l}}$	Current density vector in the electrolyte	A/m^2^
$\sigma_{l}$	Conductivity	S/m
${\nabla \phi }_{l}$	Electrolyte potential gradient	V/m
*F*	Faraday constant	C/mol
$z_{i}$	Charge number	1
$D_{i}$	Diffusion coefficient	m^2^/s
$u_{i}$	Mobility	s·mol/kg
$c_{i}$	Concentration of the ion	mol/m^3^
$\vec{u}$	Velocity vector	m/s
η	Overpotential	V
$\overset{⃑}{l_{s}}$	Current density vector in the electrode	A/m^2^
$\vec{n}$	Normal vector	
$c_{R}$	Related to the concentration of reducers	1
$c_{O}$	Related to the concentration of oxidizers	1
$\alpha_{a}$	Anodic transfer coefficient	1
$\alpha_{c}$	Cathodic transfer coefficient	1
*R*	Gas constant	J/(mol·K)
*T*	Temperature	K
$\phi_{s}$	Electrode potential	V
$E_{eq}$	Equilibrium potential of the reaction	V
*d*	Spacer thickness	m
*c_in_*	Inflow molar concentration of electrolyte solution	mol/m^3^
*u_in_*	Inflow velocity of electrolyte solution	m/s
*c_0_*	Initial molar concentration of electrolyte solution	mol/m^3^
